# Strategies for adapting under pressure: an interview study in community mental health services

**DOI:** 10.3389/frhs.2025.1719583

**Published:** 2025-12-11

**Authors:** Ruta Buivydaite, Dulcie Irving, Bethan Page, Bill Tiplady, Charles Vincent

**Affiliations:** 1 Department of Social Policy and Intervention, Social Sciences Division, University of Oxford, Oxford, United Kingdom; 2 Oxford Health NHS Foundation Trust, Oxford, United Kingdom; 3 Cicely Saunders Institute of Palliative Care, Policy & Rehabilitation Florence Nightingale Faculty of Nursing, Midwifery & Palliative Care, Kings College London, London, United Kingdom; 4 Department of Experimental Psychology, University of Oxford, Oxford, United Kingdom

**Keywords:** community mental health, pressures, adaptive strategies, short term strategies, long term strategies

## Abstract

**Background:**

Mental health services are operating under significant and sustained pressures. Healthcare professionals and managers are continually making adjustments, often improvised and inconsistent across teams. This study aimed to identify and describe the everyday pressures experienced in community mental health settings and the adaptive strategies used by senior leadership and management staff to respond. The longer-term goal is to inform more coordinated, practical approaches to managing services under pressure.

**Methods:**

We conducted qualitative semi-structured interviews with 22 senior leadership and management staff from community mental health services within a single NHS Trust in England. Interviews explored the everyday pressures encountered and the strategies adopted in response. Data were analysed thematically using a template analysis approach, guided by our previously developed framework—a taxonomy of pressures and adaptive strategies from earlier studies in surgery and intensive care.

**Results:**

The main source of pressure was a shortage of staff with the necessary skills and experience to manage increasing patient numbers and complexity. Strategies were categorised as anticipatory (implemented in advance of expected pressures) and on-the-day responses. Most were anticipatory, reflecting limited capacity for real-time adaptation. Common strategies involved controlling patient demand and prioritising urgent cases, flexing staff allocation, and enhancing staff support, supervision, and coordination. Flexible use of external services, such as primary care and voluntary sector resources, was also reported.

**Conclusions:**

Senior mental health staff employ a broad range of adaptive strategies during periods of pressure to manage patient risk, sustain patient flow, and support staff wellbeing. Compared with acute hospital settings such as surgery and intensive care, anticipatory strategies were more prevalent in mental health services, where day-to-day flexibility is constrained by staffing and service structures. There is significant potential to train clinical leaders and teams in the effective use of adaptive strategies and to improve cross-service coordination. The framework used in this study offers a shared language and a practical menu of options to help mental health services prioritise rising patient demand and safeguard staff wellbeing.

## Introduction

Health services have been under increasing pressure for many years as the result of a growing population, increasing complexity of illnesses and comorbidities, and a shortage of resources of all kinds (particularly skilled and experienced staff) (1). Mental health services are under strain due to rising demand and falling recruitment. Clinical teams adapt to these everyday pressures to minimise risk to patients and often achieve good outcomes despite adverse circumstances (2). While individual adaptations of care may be reasonable and necessary, the overall effect of multiple adaptations on the quality of care is variable (3). If pressures persist, temporary fixes risk becoming permanent, leaving a chronically stressed system where professionals cannot meet expected standards of care (4). It is therefore vital to understand these adaptations, assess their impact, and develop safe, coordinated responses. We have previously reported studies of adaptive strategies in surgery and intensive care (5, 6). Strategies used to respond to pressure show common themes but vary according to context. This paper examines pressures and adaptive strategies in UK community mental health services.

### Mental health services in the United Kingdom

In the United Kingdom the pressure on mental health services has been steadily increasing over the past two decades (7, 8). A 2010 report noted major cuts to funding of mental health services which led to large scale reductions of services and an increased emphasis on self-management by patients and families and support from community services (9). Between 2016 and 17 and 2021–22, the National Health Service (NHS) mental health workforce grew by 22%, yet referrals increased by 44% (10). Covid-19 further worsened staff shortages as many left the NHS, exacerbating the shortage of skilled, experienced staff (11, 12). These pressures have had tangible effects on the way work is done, staff experience, and patient care (13, 14).

The cuts to mental health beds have exacerbated waiting times for treatment for some of the most serious mental illnesses (15). Current estimates put the mental health waiting list at 1.2 million people (16). reflecting sustained demand that exceeds service capacity. Prolonged waiting times intensify pressure on staff, as clinicians must manage growing caseloads, respond to patients in crisis while others await care, and cope with the moral distress of being unable to provide timely interventions—all of which contribute to chronic stress and exhaustion. The mental health workforce reports higher emotional exhaustion than emergency nurses and equal burnout to cancer professionals (17). This has a significant impact on patients and staff who may experience significant moral injury through not being able to provide the standard of care they aspire to (18).

### Adaptive strategies to respond to pressures and manage risk

We previously conducted a review of empirical resilient healthcare studies to explore the nature of adaptations to care during periods of pressure and to create a taxonomy of pressures and adaptations (19). We have also examined pressures and strategies employed in surgical care and in intensive care (5, 6). Our own review and other studies (20) found that the primary source of pressure is a mismatch between demand and capacity. This pressure makes working conditions more difficult which in turn increases risk to patients and creates more pressure on staff. We found that adaptive strategies could be divided into actions taken in anticipation of pressures and actions taken on the day. The primary types of adaptive strategy, both in advance and on the day, are strategies which enable the flexible use of resources, approaches to controlling or prioritising demand and adaptations to the way care is delivered. We suggested that knowledge of this framework and the associated strategies could enable clinical leaders to develop a coordinated approach for teams to deliver safe care and service efficiency when pressures are high (19).

The aim of the present study is to explore the everyday pressures experienced in community mental health services and to identify ways in which community mental health teams adapt clinical practice to meet demand while managing risks to patients. We use our framework of pressures and adaptive strategies to explore in depth how community mental health teams are adapting to everyday pressures and to describe the adaptive strategies they use to respond. In the longer term it may be possible to define a portfolio of adaptive strategies that clinical leaders might employ and evaluate in their own mental health services.

## Methods

### Study design

We conducted 22 semi-structured interviews with senior staff from 15 teams working in adult community mental health services in an NHS Trust in England to identify the types of everyday pressures experienced, and the adaptive strategies they use to respond to these pressures.

This project was reviewed by the Oxford University Research Ethics Committee and Oxford Health NHS Trust Research and Development Board and assessed by both as a service evaluation. As such, it was not subject to the Department of Health's UK Policy Framework for Health and Social Care Research (2017) and did not require a full ethics review (21).

### Sampling and setting

In the United Kingdom a community adult mental health team provides mental health support and services to adults within a specific geographical area, aiming to deliver care in the community rather than in hospitals. These teams are part of the wider mental health services offered by the NHS and local authorities, and they often include a range of professionals like psychiatrists, nurses, social workers, and therapists. These teams respond to a wide range of mental health problems and provide a full range of services covering assessment, diagnosis, treatment, crisis support and often more general programmes to enhance mental health in the community. Teams also engage widely with other services such as primary care and social care.

The Trust where the study took place has 8 adult mental health services, 5 services for older adults, a social care mental health service together with a gateway service which provides a single point of access for mental health services. We approached 29 members of community adult mental health teams with 22 agreeing to be interviewed: Service Managers (*n* = 2), Team Managers (*n* = 10), Deputy Team Managers/senior nurses (*n* = 2), Consultant Psychiatrist (*n* = 6), Consultant Psychologist (*n* = 1) and Allied Health Professionals Lead (*n* = 1). Data collection was completed between April and August 2024. We transcribed and carried out preliminary analysis of the data during the study, continually monitoring emerging themes. After interviewing 22 participants, the research team agreed data saturation had been reached as no new themes were emerging.

### Data collection

The interviews were informed by the findings from previous studies and our recent scoping review on adaptive strategies in healthcare and adapted for this context (5, 19). The interview codebook was iteratively developed with the aim of understanding the pressures staff faced and collating the strategies that individuals, teams and organisations use to adapt in response to everyday pressures (Supplementary File 1). The semi-structured interview guide was informed by, but not identical to, the framework and associated codebook. The interview questions were designed to elicit participants' experiences of organisational pressures and adaptive strategies, providing data that could subsequently be mapped onto, or extend, existing framework categories. Participants were invited to take part via email and sent a full information sheet. Verbal consent was obtained at the beginning of each interview, which included permission to record the interviews for the purpose of generating a transcript. Semi-structured interviews were conducted over video call, audio-recorded and transcribed verbatim. Field notes were taken during the interviews to follow-up on points of interest or for clarification. Each interview lasted approximately 1 h and there were no repeat interviews. Data saturation was achieved after 18 interviews, when no new codes or themes emerged from the analysis. The remaining four transcripts were reviewed to confirm thematic stability and to ensure that all relevant variations in experience were captured.

### Analysis

**Figure 1:**
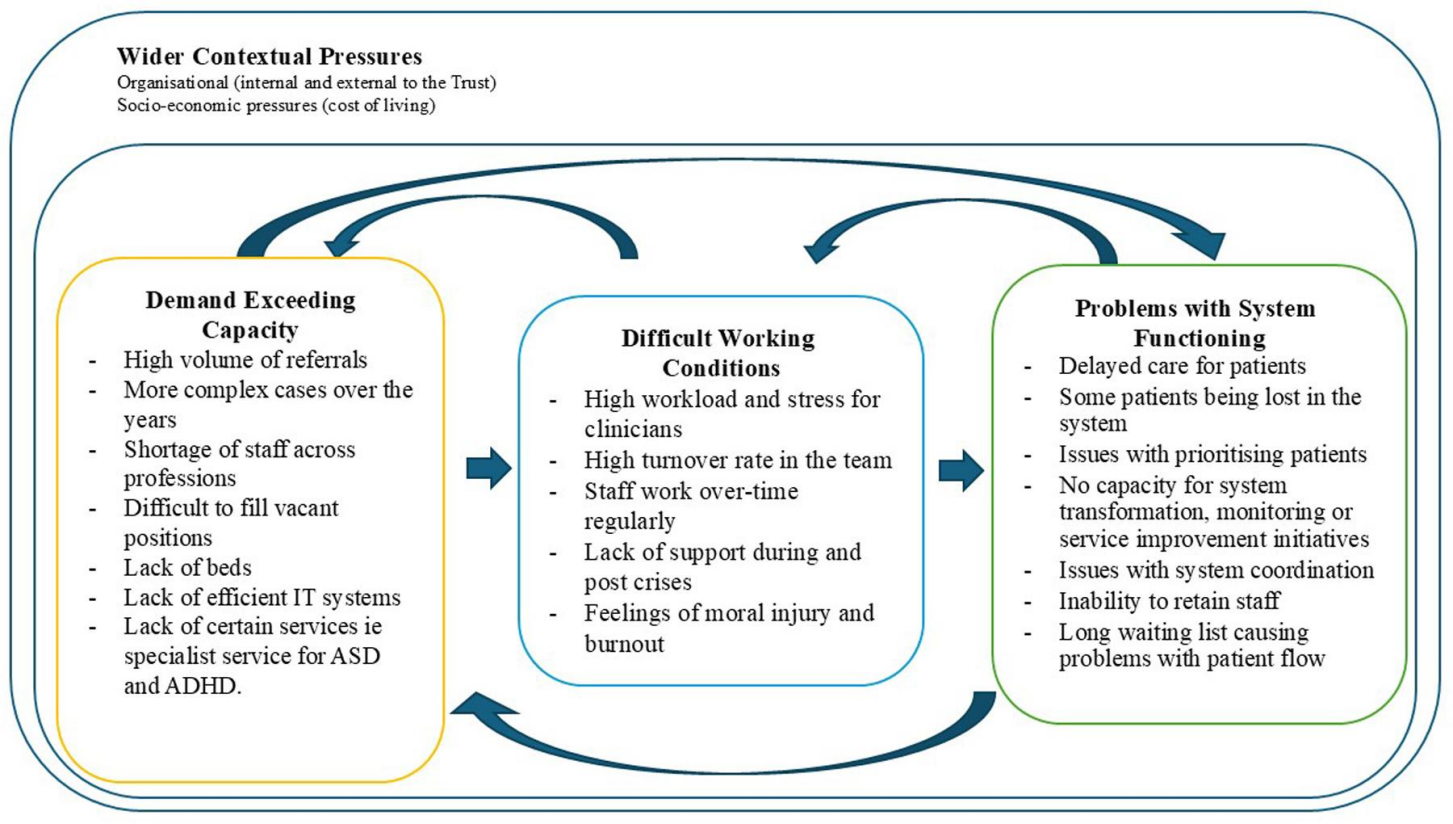
Current pressures in mental health services.

The data were analysed using a thematic template approach (22), supported by the qualitative data management tool NVivo. In the first stage, RB and DI familiarised themselves with transcripts, sharing initial reflections and coding the first transcript together, before coding the rest independently. The second stage was to create and iteratively develop a framework for organising the data, drawing on the interview guide and the taxonomies of pressures and strategies developed from the scoping review conducted by our team (19). These taxonomies provided a useful framework for organising the pressures and strategies described in the interviews and formed the major theme headings for the analysis. The framework and associated taxonomy of factors has been previously found to successfully capture the key pressures and strategies described in similar studies conducted in acute hospital settings (5, 6). However, an inductive approach was also maintained to ensure openness to new and unanticipated themes emerging from the data. Any insights that extended or challenged the framework were discussed within the research team and integrated into the evolving interpretation. For example, Figure 1 (below) depicts the refined framework of pressures and adaptive strategies. It integrates findings from this study with elements of the pre-existing taxonomy, illustrating how leadership and management responses are operationalised within community mental health settings. Data from each transcript were then indexed through systematically coding quotations and placing them in one (or more) of the framework categories. All major categories were represented, except *for flexing the resources through stopping the services* and *managing the beds or medical equipment*, which were not relevant to the community mental health context examined. Completion of these steps provided a manageable data set to analyse. RB and DI led the data analysis, and CV provided oversight. The research team met regularly to discuss the analysis and coding framework.

### Reflexivity statement

Research team members interviewing study participants considered the ways in which their interactions with participants might be influenced by their own professional background, experiences, and prior assumptions. Throughout the analysis, the research team maintained a reflexive stance, acknowledging the potential influence of the pre-existing framework while actively seeking to identify data that introduced new or divergent insights beyond it. RB and DI interviewed all the professionals. RB has 12 years of experience working in research and is also a qualified clinical psychologist and DI is an experienced researcher and a trainee clinical psychologist. RB and DI had regular meetings throughout the analysis process to develop the themes. CV, DI and RB all have experience working or conducting research in adult mental health services. The findings were shared with CV and BP who are experts in the patient safety research and have authored papers on the system adaptation under pressure. BT, who represents the Trust, provided context and feedback on the results and the paper.

## Results

The analysis confirmed the relevance of the established framework of pressures and adaptive strategies while also revealing several refinements specific to mental health services. These refinements included greater emphasis on relational and emotional aspects of staff adaptation, the importance of inter-team collaboration, and the integration of third-sector partnerships as a distinct organisational strategy. We report the findings in three broad sections, corresponding to the main categories of our framework of pressures and strategies. We first report the pressures described by interviewees, followed by the anticipatory strategies used to manage these pressures and then the on-the-day strategies.

### Current pressures in mental health services

Participants reported rising numbers and severity of patients driven by greater awareness and willingness to seek help. A shortage of skilled staff leads to high caseloads for staff. These pressures lead to more difficult working conditions, which in turn lead to wider problems in the functioning of services (Figure 1). Rising living costs drive turnover and make recruitment, especially to lower bands, difficult. In-patient bed shortages force clinics to manage high-risk patients for longer while they wait for a bed to become available. Closures of specialist services and inadequate IT systems further increase workload. With limited capacity, services prioritise patients with the most serious problems, which means that those with less severe conditions often deteriorate while waiting for care. These pressures affect staff well-being, often leading to burnout, moral injury and long-term sick leave, worsening staff shortages.

### Anticipatory strategies

Managers in each team had devised strategies in anticipation of increased pressure. As in previous studies we found that these can be classified as (i) increasing resources, (ii) controlling demand and (iii) plans for managing the workload (Table 1, Box 1). Adjustments of this kind are always necessary in day-to day work to some degree. The focus in this study, and in these findings, on those adaptations are particularly prominent during periods of pressure.

**Table 1:** Anticipatory strategies used in CMHTs with illustrative examples.

Theme	Anticipatory strategy	Example
Increase resources		
	Creating new roles to triage referrals more effectively	This new role enabled referrals to be screened and triaged within primary care which significantly reduced the number of assessments conducted by AMHT.
	Recruit below the appropriate grade and develop skills	Many interviewees spoke about the difficulty of recruiting experienced nurses. A common strategy was to recruit nurses at a more junior level in a development role and then supporting them in the role with the skills and knowledge to enter a more senior role in a few months' time.
	Enhance role flexibility	One team was improving inductions by having all new starters spend time shadowing other team members and understanding their roles so that they could more effectively cover them at times of staff shortages.
Control demand		
	Triage more effectively	All teams attempted to triage more effectively from the start to keep the caseload in check. This involved stricter application of referral criteria and signposting patients to other services when they did not meet the criteria
	Additional triage training for staff	More triage training in the application of stricter referral criteria was being provided to junior staff who were responsible for initial triages and decision on referral.
	Early discharge	At times of intense pressure it was necessary to discharge patients earlier than desired and refer them for support from the GP or another service. Supervision was often used as a check-in for identifying patients who could be discharged safely in order to maintain patient flow.
	Suspending or restricting services or procedures	When pressure or referrals and acuity is high, anything non-emergency or routine was cancelled. One team also capped the number of cases for key workers, which meant they developed a long waiting list but the levels of stress in the team were better managed.
Plans for managing the workload		
Efficiency strategies		
	Anticipatory prioritisation	Traffic-light systems were used to prioritise how often patients were seen based on their acuity, and indicate possible cases for early discharge
	Flexible working and cover between teams	Agreements had been made to expand cross cover at times of pressure by making arrangements with other teams in the region.
Monitoring and coordination within team		
	Additional briefings to cover patients in crisis	Additional short briefing meetings would be instituted at times of high pressure to discuss patients who were in crisis or needed additional support. These additional meetings also supported staff in managing caseloads and sharing risk and responsibility.
	Planned coordination across other teams and services	Regular meetings with other teams/services on a regular basis in order to coordinate referrals, waiting lists, raise any concerns and provide consistent care to patients in different localities. Good liaison with primary care services was a key for teams to anticipate and prevent future pressure.
	Support from the third sector	Establishing good relationships with charities and other third sector services supported earlier discharge of patients to the community.
Staff support initiatives		
	Encouraging a good work-life balance	Team leaders included encouraging annual leave, breaks and going home at the right time.
	Guidance for managing caseload	Supervision meetings were vulnerable to cancellation at times of pressure. The more limited supervision available focussed on providing support to staff with managing their caseload, discharging patients and helping them resolve issues swiftly during times of high pressure
	Settling limits on direct clinical work	Some interviewees in leadership roles set boundaries on the everyday clinical tasks assigned to them because they wanted to allocate more time and resources to their primary responsibilities such as managing the team and improving service delivery.

BOX 1 **Examples of anticipatory strategies.**

Early discharges to reduce caseload

“I met with each different consultant to do a caseload review of every single patient and their caseloads, to see who we can identify for discharge, who might not be quite ready for discharge, but who can we discharge? What's the barriers to discharging them? Just so that we could try to ease some pressure.” [Team Manager]

Restricting services, reducing intensity and frequency of appointments

“I think the only things that are you know not lifesaving, that's got to be reduced. I used to do a lot more kind of ADHD, ASD kind of work. That's definitely reduced. And also, you know you try and see people a bit less often when you have to, so you see a bigger volume of people, but you see them at lower intensity.”—(Consultant Psychiatrist)

Improving efficiency by utilising technology more effectively

“I realised that we had clinics in three separate locations, and each clinic diary was being kept in a different way…. the booking of patients was quite haphazard. All three clinics are now kept on one Outlook calendar and colour coded. All the clinicians provide slots which they would have to commit to … So there doesn't have to be any kind of discourse, are you available this day? Can you do this, can you do that” (Older Adults Team Manager)

Expanding team roles and skills

“What I'm trying to do is actually to up-skill all the staff to be able to do both, to have both a small treatment caseload and to be able to assess brand new case- patients so that they can slot in, in sickness, so they can cross cover, so that they can be more useful to the wider team as and when necessary.” [Team Manager]

Coordination with other teams to manage crisis situations

“We have agreements with other teams. So for instance, if we got a whole bunch of urgent referrals and we just did not have the staff to do that, the crisis teams have agreed to help us out on those occasions…. So, we were able to kind of pre-empt some of that by preplanning.” [Team Manager]

Liaising with GPs to manage the number of referrals received by the team

“A lot of good liaison with Primary Care, so we've started attending Primary Care meetings. I attended one once a week and we can immediately rebuff so many referrals because what the questions they're asking that would have led to a referral can be solved within a couple of minutes conversation. So they're really helpful” [Deputy Manager]

#### Increase resources

The shortage of permanent staff meant that reliance on locum and agency staff was one of the principal strategies for maintaining the service. Some clinics would also anticipate shortages and pay for a shadow shift, so that when a staff member went off sick, they had a pool of trained staff to draw on.

Innovations in staffing led to increased efficiency of some processes. For example, a new role had been created to triage and respond more quickly to primary care referrals. These workers were located in general practice surgeries and able to screen out and redirect inappropriate referrals and so reduce the number of assessments carried out by CMHT teams. Finally, diversifying the workforce from the start allowing the new staff to shadow different roles in the team so they can provide cover for other roles when needed.

#### Control demand

Several strategies were in place to control, and in some cases limit, the number of referrals coming into the clinic. Referral criteria were enforced more strictly to control demand and signpost patients to other services where appropriate. Routine assessments and treatment were frequently cancelled to focus the resources of the team on emergencies. Additional triage training was also provided to other staff members to improve efficiency during busy times.

At times of very high-pressure teams discharged patients earlier than was desirable and relied on the GP or third sector (charity) services to support patients after discharge. One team also assessed the overall caseload, both numbers and acuity of each key worker, and imposed a cap on individual workload. This meant they developed long waiting lists but the levels of stress in the team were better managed. Services are always controlling demand to some degree, but these strategies provide additional control and flexibility at times of increased pressure.

#### Plans for managing the workload

In order to manage the high referral rate across clinics, teams developed more rapid means of communicating the status of patients and caseloads on a regular basis. For instance, due to the hybrid working in one team, they had installed a virtual board which updated daily so that all staff would know who is where and who is available so that the team could adapt where needed and know who was available to respond to crises on the day. All teams had duty cover arrangements for urgent, and crisis work, as standard practice. However, some managers had extended this to wider regional cross-cover by making arrangements with other teams in the area with the aim of enhancing the overall resilience of the system.

Initiatives to provide support to staff wellbeing were a critical strategy for planning for managing at times of high pressure. Prioritising well-being of staff and setting boundaries to limit the amount and nature of work to be done were considered vital for the service. Managers set aside time to manage their primary responsibilities and improve service delivery, but this necessarily meant they were less available to deal with emergencies. Some teams had introduced additional support services from psychologists to support staff in prioritising and managing their caseloads. The support was not simply in planning and organisation but for the stress of not providing the care that patients needed. In all teams there was a strong emphasis on attempting, where possible, to share the responsibilities of caring for high-risk patients who might harm themselves or others.

### On the day strategies

Strategies for use on the day fall into the same broad overall categories as anticipatory strategies. The options are broadly to (i) flexible use of resources, (ii) prioritise demand or (iii) adapt ways of working. However, there is some overlap between actions taken in anticipation of problems and the more immediate adjustments on the day. This section highlights those strategies that are particularly critical to managing unfolding and rapidly changing clinical work on the day (Table 2, Box 2).

**Table 2:** On the day strategies with illustrative examples.

Theme	Strategy	Example
Flexible use of resources		
	Flexing staff for emergencies	In emergencies all staffed would be pulled off their planned work to help manage the situation.
	Rely on staff resilience and commitment	Across teams and bands, staff regularly worked overtime.
Prioritise demand		
	Prioritisation according to patients	Priorities of the service meant that patients most at risk or who had been waiting longest. For example, patients from the memory clinic were de-prioritised in favour of adult patients who were at risk.
	Prioritisation of work and workload	Sacrifice or reduce routine, but often essential, activities such as quality of assessments, level of supervision, reduction in availability or not attending routine meetings.
Adapted ways of working		
Adapted communication		
	Increased use of *ad hoc* meetings	When pressures were high, teams would often increase their level of both virtual and in-person communication. *ad hoc* meetings would also be called by senior leadership during times of pressure.
	Tools for monitoring and communicating information	Teams relied increasingly on boards to update on the status of the service and which staff were available to respond to crises.
Adapted leadership		
	Greater presence and visibility	Leaders increased their presence and availability. They were present in all the team meetings and huddles and encouraged direct emails or face to face meetings from any member of staff.
	Providing more support to staff	Additional brief check-ins throughout the day along with debriefs after difficult days. A space to was provided to staff to help them discuss and cope with traumatic events.
	Sharing risk and responsibility	Creating a team culture to share concerns with each other and have mutual support to manage difficult cases. This ensured that risk was shared among team-members, and staff feel supported and equipped to provide the best level of care.
Adapted Teamwork		
	Clear role allocation	Team leaders were more didactic and direct during times of pressure with clear allocation of roles and responsibilities.
	Utilising networks	Some managers had built up extensive network of organisations, particularly charities, to provide guidance, support and additional resources.

BOX 2 **On the day strategies.**

Staff work late regularly to accommodate high caseload

“I suppose a way that the pressures are dealt with is by staff working in excess of their hours. Is that what you mean? I would be fairly confident to say that every one of my managers probably put in between 10 and 20 h extra a week. To be fair, it's not just the managers either, it's the key workers. Most people do significantly over their paid hours. I think if it was one of the things that the NHS was to go, no, nobody can work overtime; not that they get paid for it, but you can't do that, it would really show a significant shortfall in capacity.” [Service Manager]

Prioritisation of cases according to acuity during times of high pressure

“Yes, I mean I guess the prioritising people who are at risk, risk of self-harm, risk of aggression to others, those kind of things need to be dealt with straight away whereas often it's the Memory Clinic work that gets pushed to one side because that tends to be not urgent in terms of assessing and diagnosing dementia and starting medication, that's less urgent but I do still try and make time for that.” (Consultant Psychiatrist)

Providing support and supervision to team to manage difficult cases

“It's about working within teams and feeling that you're not going to be blamed for things if they do go wrong. It's feeling that risk is managed as a group, as a team, it's not just you are holding it. That if you go and see a risky patient, then you don't go home on your own thinking about that patient. That actually there are people there for you to discuss that patient with who are sharing that risk with you and helping you put plans in place for how that patient's going to be supported when you're not around… So a lot of it is about the relationships within the team and making sure the structures are in place within teams that support people and enable people to feel supported.” [Service Manger]

Using networks to advise and help

“So having a good leadership team and having good networking across the Trust, I think, so having other team managers, service managers, colleagues that you can lean on for advice, so you're not isolated in the pressures of the job is really important. I will often talk to my counterparts in [Region], and I've also built some bridges with my colleagues in other area … so I will lean on them as team managers and say, “How are you doing this? How are you managing this pressure? What have you done?” and sharing advice and initiatives.” [Team Manager]

#### Flexible use of resources

Mental health has limited means of flexing resources on the day. The main approach was simply working harder and longer to manage the high workload. During emergencies, staff could be taken from routine work to help manage the crisis situation, adding to the workload further. For instance, managers would step in and take on clinical roles and responsibilities to compensate for staff shortages. In addition to the burden on individual staff, this means that there is little resilience in the system to managing additional demands such as unexpected illness or absence.

#### Prioritisation of workload

Prioritisation of patients according to need is of course an ordinary feature of clinical work but takes on an increasing important during periods of pressure. Teams increased the frequency of meetings to assess the urgency and acuity of new referrals and rescheduled appointments of patients who were at less risk. Safeguarding incidents, for example, would take precedence over routine work, and planned supervisions or meetings would be cancelled. However, this strategic prioritisation of patients most at immediate risk, led to a neglect of relapse prevention work with others, which in turn could create pressure down the line. In the short-term this is a reasonable strategy but can lead to a long-term reduction in supervision, training and other core activities.

#### Adapt ways of working

Adaptations were made to communication, leadership and teamwork during periods of pressure. Team members would communicate more frequently but also more briefly. Leaders would call ad-hoc meetings to monitor and adjust to unfolding pressures. For instance, in one team, the duty desk was a hub where consultants and managers would congregate to talk about the situation and troubleshoot together in person. Virtual mediums like WhatsApp and MS Teams were also used to facilitate quick communication internally in the team.

Leaders increased their presence in team meetings and huddles and had multiple ways for staffs to approach them at any time, either face to face or via emails. Additional debriefs at the end of a difficult day were encouraged and after any traumatic event a team debriefing session would be organised, allowing the team to talk about it and support they need. Managers became more insistent on staff taking annual leave, breaks and going home at the right time.

While teams endeavoured to take decisions collaboratively, during periods of pressure senior staff often had to take decisions without consultation of discussion simply because of shortage of time. Many tasks were delegated to administrative support staff, and lower band clinicians, to enable managers dealing only with the most pressing matters. While this is an efficient and desirable management approach, during periods of pressure junior staff sometimes had to take decisions which fell well outside their usual responsibilities. The overall pattern was that high pressure reduced information sharing and collective decision making.

## Discussion

The principal source of pressure identified in community mental health, as in other settings, is a shortage of staff with the necessary skills and experience to be able to cope with the increased numbers and complexity of patients. These pressures produce difficult working conditions, which in turn have wider effects on patient flow and the functioning of the system overall. Senior leadership and management staff deploy a portfolio of strategies to respond to these pressures: most strategies are deployed in advance of anticipated pressures and some on the day to manage immediate pressures. Senior leadership and management staff described a wide variety of strategies to increase or flex resources, control or prioritise demand and adapt the way in which care is delivered. In spite of the very different nature of the services studied the framework of pressures and strategies was as relevant to mental health services as it was to intensive care and surgery.

Coordination across services emerges as a strength in mental health care, distinguishing it from settings like ICU or surgery. The reliance on teamwork—both within and between teams, including third sector partnerships and closer working with primary care—helps distribute risk, manage patient demand and support staff. Strategies for patient prioritisation and case load allocation are critical for making the most effective use of the available staff resources, prioritising the patients most in need whilst not overburdening staff (23, 24).

Clarifying referral criteria and caseload weighting (e.g., green, amber, red classifications) offer tangible solutions to manage workload fairly and improve service flow: these are vital for enabling leaders to maintain oversight of workload and patient risk particularly during periods of pressure. Similar strategies were also identified in previous studies that highlighted the importance of actively managing the caseload in mental health settings (25, 26). These efforts parallel gatekeeping strategies used in intensive care but are tailored to the unique demands of mental health services team (6).

There were many parallels between the pressures and strategies identified in this study in community mental health and our previous studies in surgery and intensive care (5, 6). While the framework of pressures and strategies was applicable across all three service contexts studied, notable differences were observed in how these strategies were enacted. In intensive care and surgical settings, adaptive responses often centred on immediate operational control, rapid escalation pathways, and highly structured team coordination to manage acute surges in patient demand. In contrast, mental health services operated within a more diffuse, longitudinal model of care, requiring sustained interprofessional collaboration, negotiation of caseload boundaries, and attention to relational continuity. These distinctions underscore the need for context-specific approaches to workforce resilience and service adaptation.

The findings also highlight several features unique to mental health services—particularly the relational intensity of care, the chronicity of patient need, and the sustained emotional exposure experienced by staff—which distinguish these settings from acute physical health environments. These contextual factors appear to shape the ways in which resilience and adaptive responses are developed and enacted within mental health teams.

The increasing pressures within mental health services have heightened awareness of the critical importance of supporting staff wellbeing. These pressures arise not only from the inherently complex and emotionally demanding nature of mental health practice but also from the growing frequency with which professionals encounter systemic constraints that prevent them from delivering care consistent with their personal and professional standards (27). In this context, organisational leaders must implement structured and anticipatory strategies to support staff during periods of sustained operational strain, including explicit acknowledgment and discussion of the ethical and practical trade-offs involved in clinical prioritisation. Mental health practitioners are frequently required to focus resources on those in acute crisis, necessarily delaying interventions for individuals assessed as lower risk or more stable. Such delays can inadvertently contribute to the deterioration of patients' conditions and generate moral tension among practitioners. Although short-term departures from ideal standards of care are often regarded as unavoidable within constrained systems, their persistence over time can engender significant moral distress and psychological harm, as clinicians struggle to reconcile their professional values with systemic limitations (28).

### Tensions between short-time adaption and long-term impact

Although this paper has focussed on short term strategies for working under pressure, there are of course many plans and initiatives to improve both recruitment and retention of staff in the longer term. The challenges of recruiting sufficient staff are considerable and shortages are likely to be experienced for many years to come. The reallocation of skilled staff into higher bands may resolve immediate gaps but inadvertently intensifies strain on mid-level staffing, further highlighting the importance of long-term planning over reactive fixes (27, 29).

A recurring theme is the tension between short-term adaptations and their long-term impact. Stopgap measures such as employing locums, remote working, or management stepping into clinical roles address immediate needs, but risk exacerbating workforce instability and leading to burnout if normalised (30). The system's current dependence on goodwill and adaptability, however, creates a feedback loop where staff feel exploited.

### Training in adaptive strategies and support for working under pressure

The strategies described here, if explored more fully in similar environments, could help clinicians and managers respond to similar pressures, providing a portfolio of co-ordinated strategies that clinical teams could use or develop for their own contexts. For example, scenario-based teaching around managing competing demands could help to reduce stress and uphold safe practices when individuals have to make strategic decisions quickly in pressurised situations (31). There is also scope for exploring wider organisational changes. In surgery, for example, many hospitals now separate elective and emergency operations to avoid the continual disruption of routine work. Emergencies and urgent care are managed by separate teams allowing routine work, which forms the bulk of the services, to be planned and predictable and so more efficient. Individual mental health teams might struggle to implement this model, but it could be explored at an organisational or regional level.

In addition, there may be lessons to draw from scheduling strategies in surgical services: for example, a quality improvement audit of elective general surgery in the UK found that 22% of scheduled procedures were cancelled, with many cancellations occurring within 48 h and due to patient illness, administrative issues or unpreparedness (32). Techniques such as maintaining standby lists, reviewing patient fit immediately prior to surgery, and checking administrative readiness were among the proposed interventions. These approaches might offer useful parallels for community mental health settings in managing waiting lists and prioritising patients dynamically.

There may be important learning by bringing together leaders from different clinical settings, to discuss the strategies used to adapt to pressures. For example, there may be parallels in scheduling for efficiency and patient prioritisation between mental health and surgery (5) or strategies for managing staff wellbeing or early discharges between community mental health settings and adult intensive care (6).

Wider, more formal training programmes will require organisations, and indeed regulators, to explicitly acknowledge the difficulties of maintaining standards of care when under pressure and see such training as a necessary form of proactive risk management (33, 34). Future work will explore what this type of training might look like and how it could be organised, with attention given to efficacy, trade-offs and implications of a menu of strategies.

There is a critical role for leaders, both executive and clinical, in discussing such compromises openly and supporting teams faced with unenviable decisions. The risk of moral injury will be less if such decisions are seen as a necessary collective decision rather than an individual personal failing (18, 28, 35).

### Policy context

Recent national initiatives highlight the wider context in which our findings sit. The government has announced a refreshed NHS workforce plan aimed at tackling ongoing staffing shortages through new training and retention strategies (36). Alongside this, NHS England has sought to streamline priorities in its 2025/26 operational planning guidance, granting local systems greater flexibility in addressing population needs (37). Structural reforms, including the consolidation of NHS national bodies, have been framed as a means of reducing bureaucracy and redirecting resources to the frontline, though their effect on staff morale and capacity remains uncertain (38). Efforts to expand virtual wards, diagnostic hubs, and digital services are similarly intended to relieve hospital pressures but introduce fresh demands for skills and training (39). Evidence from the COVID-19 pandemic shows that redeployment and rapid role changes left a lasting impact on staff experiences and service delivery (40). Moreover, workforce organisations such as UNISON continue to emphasise that retention and staffing concerns cannot be solved through productivity measures alone, but require sustained investment and planning (41). Together, these national-level developments demonstrate both recognition of the crisis and the continuing tension between policy ambitions and the realities of service delivery.

The results offer practical guidance for other NHS Trusts and comparable service contexts by demonstrating how senior leaders can adopt anticipatory and system-level approaches—such as proactive workforce planning, structured staff wellbeing initiatives, and strengthened collaboration with primary care and voluntary sector partners—to manage sustained pressures more effectively. Specifically, the findings highlight the transferability of adaptive strategies, which can inform service improvement initiatives across diverse organisational settings. Together, these national-level developments demonstrate both recognition of the workforce crisis and the continuing tension between policy ambitions and the realities of service delivery. Importantly, the framework developed in this study provides a practical tool to guide the implementation of these adaptive strategies, supporting leaders to plan, coordinate, and evaluate responses to workforce pressures across the wider mental health system.

### Strengths and limitations

Our choice of interviews for data collection meant that participants could discuss and reflect on adaptive strategies, which methodologies such as ethnography used in other similar studies are less suited to; conversely there may be some strategies that could be observed through ethnographic methods but are not easily describable by participants in interviews which we may have missed. We were also not able to evaluate the effectiveness or impact and unintended consequences of the various strategies which needs to be a priority for future research.

While the findings are contextually grounded in one NHS Trust, the identified strategies are likely to be transferable to similar community mental health settings. Variations in local governance, resources, and workforce configuration may, however, influence their implementation. Future studies should examine a wider range of sites to examine both the extent of adaptations and how they vary across teams and organisations. As the participant sample comprised senior leadership and management staff, the study does not capture the direct experiences of more junior frontline clinicians; senior staff have more autonomy to make adaptions at a senior level but of course more junior staff do make adaptions too that could be explored in future research.

### Future research on adaptive strategies

The taxonomies of pressures and strategies previously developed by the research team (19) provided an effective framework to categorise the data from this study: the framework was able to capture the key strategies and pressures described by participants. However, the nuances of mental health work—its collaborative ethos, reliance on external partnerships, and emotional labour—should inform further refinement of such frameworks. There may be certain strategies or combinations of strategies that are better than others or have differential trade-offs and impact on safety, staff well-being, patient flow and patient experience (4, 42). A strategy may for instance reduce risk for patients but increase burden on staff: this could be explored further through vignette-based studies (43). There is also a need to assess the benefits and risks of specific adaptive safety such as enhanced triage and gatekeeping and early discharge (44, 45). However, there is also a risk of over-burdening families and exporting risk from clinical settings into the home.

## Conclusion

Community mental health teams used a wide variety of adaptive strategies to maintain patient flow, manage risk and protect staff when services are under pressure. While these adaptive strategies are necessary and aimed at providing better care in the short term, they also potentially involve risks to patients and longer-term degradation of standard of care. Our aim in this and related studies however is not simply to point to the adaptations but to pave the way for a more open, transparent, coordinated and time-limited approach to adaptations. We believe that patients will be safer if we develop prepared and coordinated strategies where the team has agreed an approach for managing risk under pressure (4).

## Data Availability

The original contributions presented in the study are included in the article/Supplementary Material, further inquiries can be directed to the corresponding author.
