# Supplementary material for: Strategies for adapting under pressure: an interview study in community mental health services
Source: Front Health Serv. 2025 Dec 11;5:1719583. doi: 10.3389/frhs.2025.1719583 (PMC12738820; doi:10.3389/frhs.2025.1719583)
Supplement: Supplementary file 1 [file Table1.docx]

**Adaptive Strategies in Community Mental Health Services Codebook**

| Codes (primary, secondary & *tertiary*) | Description |
| --- | --- |
| Context |  |
| About the interviewee | General information about the interviewee including job roles and responsibilities, as well as work background and history (e.g. nursing, prior services). |
| Descriptions of the clinical setting | General information about clinical setting the interviewee works in, including size, space, patient population, staff numbers etc. |
| PRESSURES |  |
| Demand exceeding capacity | Where patient demand exceeds the capacity of resources available. |
| Patient demand |  |
| *High patient acuity* | High severity of condition, which might include degree of being unwell or complexity of patient (e.g. comorbidities). |
| *High volume of patients* | High numbers of patients. |
| *Patients' and families' concerns & expectations* | This relates to any concerns and expectations from patients and their families related to what their care is and what they expect it to be, as well as any emotional burden taken on or complaints/bullying |
| Resource pressures |  |
| *Issues with IT systems* | Issues with IT systems, include fit for purpose, coordinated systems between teams and services |
| *Lack of correct services* | Includes codes relating to being unable to provide the correct care because right service unavailable in the region (e.g. ASD, ADHD). |
| *Lack of space or space not fit for purpose* | This code relates to lack of space to deliver desired care for patients, work efficiently or staff to do managerial/office work. |
| *Shortage of bed availability* | Shortage of physical beds or staff available to staff those beds. |
| *Shortage of medications, supplies, equipment* |  |
| *Shortage of staff numbers* | Shortage of staff numbers due to sickness, absences, vacancies, strikes etc. Might include uncertainty of staff numbers when it comes to assigning staff or staff rotas. |
| *Shortage of staff with necessary skills* | This would include levels of seniority (e.g. shortage of senior nurses) or issues with skill mix. |
| Difficult working conditions |  |
| Staff-team dynamics | Working dynamics and interpersonal relationships between staff and within teams. |
| *High staff stress or poor wellbeing* | Includes codes related to high stress, staff burnout, low morale or moral injury. |
| *Interpersonal difficulties* | Interpersonal difficulties might include codes relating to disagreements about patient care, prioritisation or ways of working, as well as individual personalities and team working. |
| *Lack of support for staff* | Lack of support for staff might refer to unsupportive management/Trust with pressures or implementing changes or simply not enough staff able to support (e.g. senior staff to provide supervision to junior staff). |
| *Team changes* | Refers to changes in the team which affects team dynamics or creates pressure, e.g. due to unknown skillset of the person. This might include high staff turnover or use of temporary staff. It might also relate to pressures of last minute changes to bridge gaps in staffing. |
| Workload pressures |  |
| *Difficulties in prioritising workload* | When the workload is so high that all tasks are just as urgent so it becomes difficult to be able to know what to prioritise and how. |
| *High or increased interruptions* | Multiple interruptions for ad hoc requests making it difficult to do current or planned tasks. |
| *High workload or increased complexity of workload* | High workload might include codes relating to volume of work for the same job or multiple different role responsibilities. Complexity of workload relates to complexity of patient presentation (e.g. comorbidities or need for specialist treatment). |
| *Time pressures* | Codes might include difficult performance targets, unrealistic expectations regarding time to perform a task, time-critical tasks or wait-times. |
| Problems with system functioning |  |
| Disruptions to patient care |  |
| *Delays in patients accessing or receiving care* | Delays that mean a patient is not receiving the correct or timely care they should, causing additional or more challenging work for staff. |
| *Missed care or errors* | Increased likelihood of missed care or errors causing additional stress and work for staff and in some cases leading to patient harm and investigations. |
| Overstretched system |  |
| *Difficulties monitoring the situation* | Pressures which mean situation awareness or oversight of the system becomes lost (particularly relating to senior clinicians becoming overburdened). |
| *Lack of buffer for ad-hoc requests or unplanned work* | The buffer that allows the team to respond to just in time demands and ad hoc requests is substantially lessened or even eliminated. |
| *Overcrowding* | Severe overcrowding due primarily to a lack of inpatient beds. |
| *Problems with patient flow* | Patients being held longer in a particular place or service creating bottlenecks through the system (e.g. problems triaging, discharging or admitting patients). |
| *Problems with system coordination* | Problems with communication to staff and patients about last-minute changes, delays or cancellations. Also includes codes about coordination between services (e.g. primary care). |
| Wider contextual pressures | Contextual pressures relate to factors external to the clinical team, which are affecting the care the service can provide. |
| Organisational pressures |  |
| *Pressures external to the Trust* | Organisational pressures external to the Trust coming in (e.g. directives from NHS England or financial pressures of the NHS more broadly). |
| *Pressures internal to the Trust* | Organisational pressures internal to the Trust, such as constraints of funding or pressures in other parts of the system (e.g. primary care). |
| Socio-economic pressures | These include wider pressures affecting society during the time of the study, such as cost of living crisis or strikes. |
| ANTICIPATORY STRATEGIES |  |
| Increase resources short-term (next week/month) |  |
| Repurpose space | Transforming existing space so that it is fit for purpose. |
| Improve skill-mix | Additional training or upskilling staff to perform certain tasks or take on new roles (e.g. assessments) or other ways of improving the skill mix within the team through staff rotas for example. |
| Increase staff | Recruit new members of staff (looking in the next week/month). **Use of locums/agency staff.** |
| Raise awareness of pressures to senior leadership (to request for some action) | Bringing or reinforcing pressures to management or the Trust in order to get additional support (e.g. funding for staff). |
| Control Demand |  |
| Discharging or transferring patients | Discharging patients earlier to create space for anticipated need (e.g. over a weekend) or transferring them to other teams or services. |
| Suspending or restricting services or procedures | Making the decision to suspend a service or stop/reduce doing certain procedures in order to gain control of patient demand or in anticipation of forthcoming pressures. |
| Clarifying and reinforcing referral criteria | The code refers to ‘gatekeeping’ the service to emphasise its remit so it doesn’t become a catch-all service. |
| Plans for managing the workload |  |
| Efficiency strategies |  |
| *Doing tasks ahead of busy time* | Anticipating a bottleneck or increased pressures on services and doing tasks that can be done ahead of time. |
| *Scheduling to maximise use of limited resource* | This might include scheduling of space, staff rotas or patient lists to optimise efficient use of resources. Scheduling time **working from home** instead of the office/department to minimise interruptions. |
| *Use of technology & automated systems* | Use of technology & automated systems might include having online appointments or using an app to maximise efficiency. |
| Forward planning |  |
| *Anticipatory prioritisation* | Prioritisation in advance of anticipated pressures (e.g. referring to guidance and prioritising patient by need or wait time). |
| *Contingency planning* | These are plans that are made and communicated in case a particular event occurs or there are sudden changes in plans (e.g. having a back-up plan or previously agreed plan). |
| *Creating or adapting protocols* | Creating or adapting protocols to simplify information and remind/train staff in the essentials of care. |
| Monitoring & co-ordination strategies within teams |  |
| *Having an up-to-date knowledge of resources and demand* | Monitoring the system capacity (e.g. space and staffing) and patient demand (e.g. waiting and readiness for discharge). |
| *Planned meetings for monitoring the situation and communicating plans* | Planned meetings for monitoring the situation and communicating plans within teams to coordinate the care of patients. |
| Monitoring & co-ordination strategies across other teams and services |  |
| *Centralised structures for co-ordination* | This category might include codes relating to triage or service coordinating to communicate service status (demand & capacity) and plans. |
| *Planned meetings and coordination across other teams and services* | Planned meetings for monitoring the situation and communicating plans across other teams and services to coordinate the care of patients. |
| Staff support initiatives | Initiatives to provide support for staff, e.g. psychological support, supervision. |
| *Being available/visible* | This includes having specific/planned office days where leaders are in the office in order to be more visible and available to support staff and troubleshoot problems. |
| Longer-term service improvement initiatives |  |
| Increase staff (longer-term initiatives) | Initiatives to increase staff in longer-term recruitment. |
| Create new space | For example, new builds. |
| Training initiatives to improve skill mix | Longer-term training initiatives (e.g. university courses, apprenticeships). |
| Open more services | New services introduced to meet the population needs and relieve pressure elsewhere. |
| Quality and service improvement projects | This code includes any quality or service improvement projects designed to address an identified pressure to everyday working. |
| ON-THE-DAY ADAPTATIONS |  |
| Flex resources |  |
| Flex Medicines and Equipment |  |
| *Borrowing resources from other units* | Borrowing drugs or equipment from other hospitals or units. |
| *Use of similar drugs or equipment* | Using similar drugs and equipment when what is usually used is not available. |
| Flex space and beds |  |
| *Creating temporary holding spaces for patients* | Creating temporary holding spaces for patients while patient flow issues are resolved. |
| *Placing patients or providing care in non-standard areas* | Placing patients or providing care in non-standard areas (i.e. places where the care required wouldn’t usually be given). |
| *Transferring or relocating patients based on need* | Diverting patients in less serious conditions to primary care or more complex conditions to specialist care. |
| Flex staff |  |
| *Adjustments to staff-patient ratios* | Adjustments to staff-patient ratios based on skill mix and experience or complexity/acuity of patient on the day. |
| *Flexing staff to address numbers or skill-mix* | How the staff available on the day itself are flexed to address any shortages or skill-mix issues. |
| *Managerial staff take on clinical roles and responsibilities* | Managers shedding managerial tasks for participating in clinical work to address staffing gaps or sudden increase in patient demand. |
| *Staff work late* | Staff regularly working beyond contracted hours to get the work done. |
| *Task-shifting or extension of responsibilities* | Describes strategies where staff might switch between tasks or junior staff given additional responsibilities. It might also include task-shifting between professions. |
| Prioritise Demand |  |
| Prioritisation of workload |  |
| *Prioritisation and reprioritising patients* | Prioritising and reprioritising patients based on criticality of their condition. |
| *Prioritising and reprioritising workload* | Prioritising and reprioritising workload based on where the need is and urgency of tasks. |
| *Temporarily stopping or delaying some activities or types of care* | Care or other activities that are delayed or stopped (deprioritised) for a short while to regain control. |
| Adapt ways of working |  |
| Communication |  |
| *Boards or spreadsheets for monitoring and communicating information* | Greater use/referral to boards (e.g. whiteboards or interfaces) or spreadsheets when under pressure to monitor and communicate status of the service (e.g. traffic light systems to manage individual caseloads). |
| *Increased communication* | Increasing level of communication at times of high pressure, for instance having additional ad hoc meetings. This is both within teams and with other services. |
| *More reliance on face-to-face and handwritten communication* | Using face-to-face communication with people in the close vicinity (i.e. not electronic communication) or multimodal communication. |
| *Other means of quick communication* | More reliance on quick communication (e.g. WhatApp or Microsoft Teams chat/call) to request urgent support and updates. |
| *Simplifying information* | Information communicated about patients is simplified so that only the most needed information is conveyed. |
| *Communicating pressures and responses to senior leadership* | Commonly understood as escalation, informing senior leadership (service managers) of pressures or risk and sharing concern (not holding it by themsel |
| Leadership |  |
| *Accepting pressure and doing enough* | Accepting that there is a level of pressure that is constant and doing the best job you can. |
| *Adjust and communicate goals for the system* | Communicating changes with the team so everyone has the same information. This might involve clarifying aims or referring to contingency plans. |
| *Greater presence/increased visibility* | Being more around and visible to the team to check-in and provide support where needed. |
| *Providing more support to staff* | Providing ad hoc support to staff on the day where needed (e.g. taking breaks, debriefing, checking on wellbeing). |
| *Stop operations and regain awareness of situation* | Stopping current task and stepping back from the situation to look at the whole system in order to regain awareness of the situation. |
| *Sharing leadership across the team* | More flattening of seniority levels (banding) and increased autonomy given to staff. |
| Teamwork within team |  |
| *Additional support for less experienced staff* | Extra support might be provided to more junior staff on the day (e.g. buddy systems, additional supervision). |
| *Adjusting or making clear allocation of roles* | Making clear allocation of roles so each member of the team knows what their responsibilities are, and adjusting these as needed. |
| *Increased collaboration and asking for help from others* | Increased collaboration between professions and levels of seniority within teams. Asking for help from others, shared decision and sense checking are also codes here. |
| *Increased use of checking mechanisms* | More use of checking mechanisms such as closed-loop communication to check understanding of instructions, checklists etc. |
| *More multi-disciplinary working* | More multidisciplinary working between different professional groups (physicians, key workers, team managers, psychologists etc) and ad hoc multidisciplinary discussions. |
| *Increased referral to protocols and guidance* | Increased referral to protocols and guidance available (e.g. escalation policies/standard operating procedures). |
| *Additional peer support* | Informal means of supporting each other within the team on the day to deal with pressures. |
| Teamwork with other teams and services |  |
| *Additional communication & coordination across other teams and services* | Additional communication and coordination strategies with other teams and services (external) |
| *Using networks to advise and help* | Using network of contacts across services and regions to advise and help (external to team). |
|  | |
| Main Objective | Main objective when services are under pressure. |
| Communication about pressures and strategies to patients and families | How the changes described are communicated to patients and families. |
| Impact |  |
| Impacts on patients and families | How the pressures described have affected the care/service provided to patients and families. Benefits and drawbacks of the strategies used for patients and families. |
| Impacts on staff | How the pressures described have affected the staff. Benefits and drawbacks of the strategies used for staff. |
| Impacts on the wider system | How the pressures described have affected the wider system. Benefits and drawbacks of the strategies used for the wider system. |
| Education and Learning |  |
| Education and learning for others | How the strategies learned could be shared/adopted by others. |
| How these strategies are developed | How the strategies used to manage when pressures are high have been developed. |
| How to teach these strategies | How these strategies could best be taught to people who need to deploy them. |
| Sage Advice | Additional sage advice from interviewees (i.e. words of wisdom) that isn’t a strategy. |
